# Editorial: Machine learning on understanding the epigenetic mechanisms underlying plant adaptation and domestication

**DOI:** 10.3389/fpls.2023.1236787

**Published:** 2023-07-04

**Authors:** Shanwen Sun, Xiucai Ye, Quan Zou

**Affiliations:** ^1^ College of Life Science, Northeast Forestry University, Harbin, China; ^2^ State Key Laboratory of Tree Genetics and Breeding, Northeast Forestry University, Harbin, China; ^3^ Department of Computer Science, University of Tsukuba, Tsukuba, Japan; ^4^ Institute of Fundamental and Frontier Sciences, University of Electronic Science and Technology of China, Chengdu, China

**Keywords:** machine learning (ML), adaptation, domestication, non-coding RNAs, trait, regulatory network

Plant adaptation refers to the process by which plants evolve certain traits that enable them to survive and reproduce successfully in a particular natural environment. Domestication, on the other hand, is an adaptive process carried out by humans for desirable traits (Ross-Ibarra et al., 2007). Plant adaptation and domestication are interconnected processes that are manipulated by selections. These selections can be characterized at phenotypic level and at molecular (i.e., genetic and epigenetic) levels. This Research Topic presents six original studies that developed and applied machine learning algorithms to identify epigenetic markers and proteins associated with key adaptive traits that are under selections or the traits themselves. These works can aid to uncover molecular mechanisms underlying plant adaptation and domestication, accelerate crop breeding, and facilitate species conservation.

Two articles focused on long non-coding RNAs (lncRNAs), an important component of epigenetic regulation of gene expression. Cao et al. first identified 2857 lncRNAs based on published RNA-seq data in potatoes. Then, lncRNAs in a resistant potato line that responded differentially (DELs) to *Phytophthora infestans* infections which lead to late blight disease were screened. A random forest algorithm implemented by GENIE3 (Huynh-Thu et al., 2010) was used to construct the directed regulatory relationship between DELs and their target genes. This effort allows authors to infer the role of DELs in potato resistance to *Phytophthora infestans* based on enrichment analyses.

One mechanism of lncRNAs involving in the regulation of gene expression is through their interactions with miRNA and thus modulating miRNA-mediated target inhibition. Cai et al. developed a new model to predict interactions between lncRNAs and miRNAs in plants based on sequence similarity and interaction profile similarity. Their model outperformed the state-of-the-art methods and can identify novel relationships between lncRNAs and miRNAs. It may thus facilitate the deciphering of the role of lncRNAs in plant key traits and adaptation.

Three articles focused on key traits that are crucial to plant adaptation and crop domestication. Fusarium head blight is a fungal disease caused by *Fusarium graminearum* Schwabe. It can lead to significant yield loss in cereal crops, such as wheat and barley, and reduce grain quality. Genomic prediction is an effective method to combat the disease (Dong et al., 2018). Most models of genomic prediction are statistical. Jubair et al. developed the first transformer model to predict Fusarium head blight disease. The authors think that the transformer’s ability to account for the connections between genetic markers is an advantage. Results indicate that their model outperforms current methods, demonstrating the potential of the transformer algorithm in genomic prediction. Another significance of their work is to identify top markers with a filter-based feature selection algorithm. This facilitates training the transformer model and recognizing potential disease resistance-related genes.

The easiness of peeling and fruit hardness are two important quality traits in Citrus, which are traditionally assessed by experts. Minamikawa et al. developed a method to assess these two traits using the cross-sectional images of citrus fruits based on machine learning algorithms and Bayesian networks. In this study, several machine learning tools and algorithms were used, including OpenCV and Scikit-image for image analysis, multiple linear regression (MLR) and non-linear Random Forest (RF) regression for the assessment of morphological features associated with fruit quality traits, and convolution neural networks for fruit quality classification based on fruit images. Based on explainable machine learning algorithms, authors found that the degradation area of the central core of the fruit and the seed area are two key morphological features associated with fruit quality. Overall, this automatic tool can increase the volume of fruit quality traits data and facilitate the domestication and breeding of citrus.

Phytic acid is a natural antioxidant found in many seeds, grains, and nuts. It binds with minerals hindering their absorption by the human body. Hence, reducing its content is necessary but it comes at a cost of decreased seed and seedling vigor and stress tolerance, as proven by previous studies (Shi et al., 2007; Colombo et al., 2020). DeMers et al. conducted research to uncover the underlying mechanism for this trade-off by generating regulatory networks through unsupervised inference methods on both low and normal phytic acid lines. These networks allow them to find several elements that are responsible for disrupted phosphate ion homeostasis, altered myo-inositol metabolism, and changed abscisic acid (ABA) signaling which may affect seed vigor. Overall, these findings suggest low phytic acid and seed vigor have a complicated regulatory relationship, necessitating traditional plant breeding for better outcomes.

One article is about the use of machine learning algorithms to identify plant resistance proteins which can help plants adapt to pathogens and reduce disease. Identifying these proteins is crucial for enhancing food security and increasing crop yields. Several tools have been proposed to identify resistance proteins based on machine learning algorithms. However, there is still room for improvement. Chen et al. have developed a new tool that improves upon these existing tools by using a stacking algorithm and considering the pairwise energy content of residues. This contribution enhances our understanding of how plants adapt to pathogens and assists in breeding crops with improved resistance against diseases.

## Author contributions

SS wrote the first draft of the manuscript. All authors contributed to the manuscript’s revision and approved the submitted version.
